# Processes underlying complex patterns of song trait evolution in a *Setophaga* hybrid zone

**DOI:** 10.1002/ece3.7559

**Published:** 2021-05-01

**Authors:** Jay Love, Franz Goller

**Affiliations:** ^1^ University of Utah Salt Lake City UT USA; ^2^ University of Münster Münster Germany

**Keywords:** aggression, birdsong, mating signals, reinforcement, vocal learning

## Abstract

During secondary contact between two species when hybrids are less fit than parents, mating signals are expected to diverge, while aggressive signals are expected to converge. If a single signal trait is used in both mating and aggression, then the dynamics between these two forces could influence the evolutionary trajectory of that trait. We studied such a situation in an avian hybrid zone between two *Setophaga* species, where birdsong is used in both mate attraction and territory defense. We hypothesized that song modules of the two species will show separate and distinct geographic patterns due to the influence of selective pressures for effective territorial aggression and for effective mate attraction. We conducted geographic cline analyses and playback experiments across this hybrid zone. We found an unexpected geographic pattern of asymmetric introgression of song rhythm, which may be explained by results of the playback experiments that suggest that differences in song rhythm serve a greater role in mate attraction than in territory defense. In contrast, differences in syllable morphology show little evidence of importance in mate attraction or territorial defense. Song features converge in the hybrid zone, yet patterns of trait change suggest that the song production modules may vary in their modes of development and inheritance. Syringeal motor gesturing, which gives rise to syllable morphology, shows a nonclinal mosaic pattern, suggesting that this trait may be predominantly learned. In contrast, respiratory patterning, which forms song rhythm, shows a clinal geographic transition, suggesting that this trait could be more innate. The results indicate that opposing forces act independently on song via distinct modules of the song production mechanism, driving complex patterns of song trait evolution.

## Graphical abstract

In a hybrid zone between two songbird species, we find complex patterns of behavioral trait evolution. We interpret results from geographic cline analyses and playback studies to suggest that opposing forces act independently on song via distinct modules of the song production mechanism.

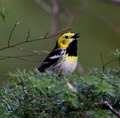

## 1 INTRODUCTION

Divergence in mating signals is expected to occur at some point during the speciation process, though the evolutionary timing of such divergence likely varies significantly according to various factors (reviewed in Coyne & Orr, 2004; Grether et al., 2017; West‐Eberhard, 1983; Wilkins et al., 2013). It is expected that such divergence contributes to reproductive isolation between populations and thus increases speciation rates compared with when divergence in mating signals is absent (Pfennig & Pfennig, 2009; West‐Eberhard, 1983). Given their clear evolutionary importance and often charismatic nature, mating signals predominate biological discussion of signaling, but signals with other functions are also used in abundance.

Aggressive signals are found widely in animals and aid in securing access to resources, including food or mates (Grether et al., 2017). While often more species‐general than mating signals, aggressive signals are also expected to diverge during the speciation process via adaptive (Endler, 1992; Morton, 1975) or neutral (Irwin et al., 2008) processes. However, unlike mating signals, aggressive signals are expected to converge upon secondary contact if genetic or ecological divergence between species is incomplete, a condition that encourages interspecific competition or territoriality (convergent agonistic character displacement; Grether et al., 2017). This phenomenon presumably arises out of selection for effective aggressive communication across species boundaries while reducing the potential costs of direct physical encounters (Cody, 1969; Grether et al., 2017; Tobias & Seddon, 2009). Convergence in aggressive signals should decrease loss of fitness due to hybridization (Grether et al., 2017), since mates and mating territories will be defended against competing members of both species.

Birdsong can be used as a mating signal and as an aggressive signal (Marler 1960, Collins, 2004; Searcy & Nowicki, 2005). Thus, in cases of secondary contact between two closely related species, we expect disparate selective pressures to act on song. In such a situation, selection for the facilitation of aggressive signaling should result in song convergence between populations, while selection for effective mate signaling should result in song divergence between populations. Changes to song upon secondary contact therefore present opportunities for dissecting these competing selective pressures.

It has been hypothesized that in cases of dual‐purpose signals, the mating function should be the primary driver of evolutionary change (Okamoto & Grether, 2013). This suggests that the songs of two species should diverge upon secondary contact when hybrids are less fit than parents. However, empirical studies have shown mixed results, with some finding upon secondary contact song convergence (de Kort et al., 2002, Secondi et al., 2003, Haavie et al., 2004, Tobias & Seddon, 2009, Kenyon et al., 2011, Laiolo, 2012) and others upon divergence (Haavie et al., 2004; Kirschel et al., 2009) or stasis (Halfwerk et al., 2016). These conflicting results are intriguing: Why would there be such disparate outcomes in seemingly similar situations?

One possibility is that the varied patterns of song trait evolution in response to secondary contact are, in part, the result of the action of the competing selective pressures described above. As was expressed in the original theoretical formulation (Okamoto & Grether, 2013), the selection imparted by the mating function of a dual‐use signal should be greater than that imparted by the aggressive function when the signal is truly simple. If, however, the signal is complex and multivariate (i.e., arising through contributions from multiple physiological mechanisms or expressed through multiple signaling modalities), then other possibilities emerge. For example, divergence in the social function of birdsong could arise, if two distinct features of song evolve divergently in response to specific, singular selection pressures. This possibility contrasts with a scenario where potentially opposing pressures exert either stabilizing or directional selection on song as a whole. Since birdsong is a complex trait, perhaps the discrepant results from past studies of song evolution in zones of secondary contact could be explained in this way.

Complicating the situation of potentially opposing trajectories of song in response to secondary contact is the fact that for all investigated oscine species, some aspects of species‐specific song develop through learning (e.g., Grant & Grant, 1996; Marler, 1970; Thorpe, 1958). It is thought that learning increases rates of trait change through a higher copy‐error rate compared with the absence of learning, and song learning has been implicated in the rapid diversification of vocal learning songbirds (Lachlan & Servedio, 2004). However, it remains difficult to form hypotheses of precisely how vocal learning influences song evolution, because details of the learning process vary (e.g., species differences in tutor–tutee relationships, timing of the sensitive period) and substantial interspecific variation exists in which features of song must be learned (Love et al., 2019),

Disentangling culturally transmitted from genetically inherited song traits would allow a more accurate assessment of song trait evolution but has proved to be difficult. A promising approach involves the distinction between three main production “modules” that must be coordinated into an integrated system of control during song production. This distinction of modules is based on the three main neuromotor mechanisms for song production: respiratory pattern, syringeal motor gesture, and central program for syllable sequence. Each of these modules contributes specific song features (song rhythm, syllable morphology, and song syntax). Critically, these modules show evidence of differential reliance on learning for their development. Both comparative and experimental studies suggest that, in many species, the rhythm of song (the coarse patterns of sound and silence, which are produced primarily by the respiratory system) is likely to be less subject to modification through learning than other production modules (syllable acoustic morphology and syntax; Marler & Sherman, 1983, Ali et al., 2013, Araki et al., 2016, Love et al., 2019, also see Lipkind et al., 2017). Consequently, distinguishing between production modules in the analyses of song across contact zones may allow for more detailed evaluation of the direction, strength, and targets of selection relative to an approach that assumes uniform learning of all aspects of song.

Hermit warblers (*Setophaga occidentalis*) and Townsend's warblers (*Setophaga townsendi*) are sister species that are estimated to have diverged in glacial refugia during the middle or late Pleistocene and reached secondary contact approximately 5,000 years ago (Lovette & Bermingham, 1999; Rohwer et al., 2001). Despite their distinct plumage phenotypes, individuals of both species are interspecifically territorial and hybridize in three small areas in the mountains of the northwestern United States where their ranges overlap (Rohwer & Wood, 1998). Importantly, hybrids between the two species appear to have reduced fitness, as they produce fewer eggs per clutch than either parent species (Pearson, 1997; Pearson & Rohwer, 1998). Therefore, we should expect reinforcing selection to act on mating signals and decrease fitness loss due to hybridization. Townsend's males appear to be more aggressive than Hermit males (Pearson, 2000; Pearson & Rohwer, 2000), and Townsend's females produce larger average clutch sizes than Hermit females. Both factors have been used as an explanation for the supposed movement of the hybrid zone from north to south, with Townsend's replacing Hermit warblers (Krosby & Rohwer, 2010; Pearson, 2000; Pearson & Rohwer, 2000). However, recent genomic evidence suggests that this hybrid zone has not, in fact, been moving as was previously thought (Wang et al., 2019). While previous researchers have stated that both species “sing the same songs” in the hybrid zone (Pearson & Rohwer, 2000), song and its role in interspecific interactions have not been assessed in detail.

Our study aimed to help determine (a) whether different song traits evolve independently or in concert in this system, (b) which selective forces act most strongly on which song trait, and (c) how cultural evolution and genetic evolution interact through song learning and production to shape song trait evolution. Our overarching hypothesis was that song modules of the two species will show separate and distinct geographic patterns due to the influence of selective pressures for effective territorial aggression and for effective mate attraction. Here, we report (a) how different acoustic features, based on two production modules, of the songs of Hermit and Townsend's warblers vary across a hybrid zone and (b) the results from an experiment investigating the social function of song modules that may partially explain the observed patterns of spatial change.

## 2 METHODS

Over the course of four consecutive breeding seasons, we recorded songs of Hermit warbler, Townsend's warbler, and hybrid males across the Olympic hybrid zone. We conducted a production‐based quantitative acoustic analysis and performed a geographic cline analysis to investigate how song traits vary across the focal hybrid zone. In parallel, we used playback of song to study the response of male and female Hermit, Townsend's, and hybrid warblers. The playback study allowed us to investigate the social function (i.e., which song features are used in mating versus territoriality) and the social value (i.e., what trait values are associated with stronger or weaker response for a given social function) of different song features across the hybrid zone.

To form baseline expectations, we first sought to establish whether song differed between species. We assessed song recordings from Hermit and Townsend's warblers from two databases: xeno‐canto.org and the Cornell University Lab of Ornithology Macaulay Library (see Appendix S2 for accession numbers). We visually inspected spectrograms of 141 Townsend's warbler and 46 Hermit warbler songs that were recorded during the breeding season away from any hybrid zone. Because the occurrence of multinote versus single‐note introductory syllables differs between these species (Morrison & Hardy, 1983), we categorized each song into one of these two groups. We then compared relative proportions of song categories across the two species and used these results to formulate our research design (see Appendix S1).

**FIGURE 1:**
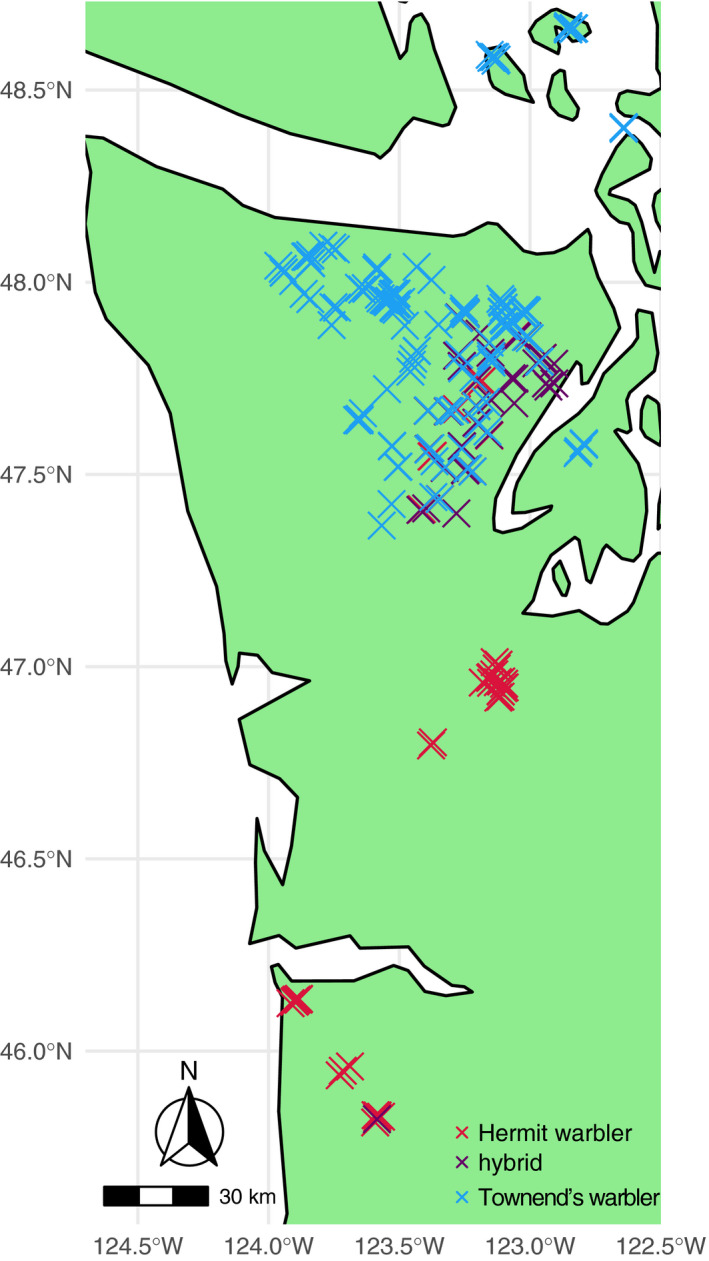
Map of study area. Crosses show sample locations. Colors indicate plumage‐based hybrid index. Red = Hermit Warbler, purple = hybrid, blue – Townsend's Warbler

From May to July 2015–2018, the Olympic hybrid zone (described in Rohwer & Wood, 1998) and surrounding allopatric regions were surveyed for singing Hermit, Townsend's, and hybrid warbler males (Figure 1). A roving approach was used to provide a random sample of individuals. Roads, trails, and off‐trail routes were followed in vehicles or on foot until a singing individual was heard; that individual was then located, and high‐quality recordings were made (Sennheiser directional microphone and Marantz PMD661 digital recorder). The individual was then attracted for identification and study using a playback speaker with the observer located over 10 m away. Geographic coordinates were recorded with a handheld GPS. Resampling was avoided primarily by tracking these highly territorial birds in the canopy and ensuring that each subsequent sample was well removed from the previous. Previously sampled regions were avoided within and across seasons.

### 2.1 Plumage scoring

Hermit and Townsend's warblers were discriminated visually on the basis of their different black and yellow plumage patterning, which was readily visible at a distance. Hybrids had intermediate plumage, and we used an established protocol for attaining a hybrid index score for individuals in the Hermit–Townsend's warbler hybrid zone (Rohwer & Wood, 1998). This identification scheme can be reliably employed in the field and represents genetic background (Pearson, 1997, 2000). Briefly, for each bird, clearly defined plumage patches (midflank, low flank, bib, face, extent of yellow on breast, intensity of yellow on breast, crown, and back color) were scored from 0 (Hermit‐like) to 1 (Townsend's‐like) and then averaged. Using binoculars and digital photography, we followed previous protocol with one exception: Face coloration was included in the overall score. This trait was omitted in Rohwer and Wood (1998) because it was considered a single‐locus trait, but subsequent research in congeners indicates that it may be a multilocus trait (Brelsford et al., 2017). Furthermore, recent evidence suggests that in these warblers, face color is tightly genetically linked to crown and breast coloration (Wang et al., 2019). Since our dataset includes many individuals with intermediate cheek patch coloration, we feel confident that including all measured plumage patches in our scoring system contributes positively to our ability to infer genetic background.

### 2.2 Acoustic analyses

From each recording, the single song with the greatest acoustic quality was selected for analysis (high signal‐to‐noise ratio) and extracted as a separate short.wav file using Praat software. Each short file was band‐pass‐filtered between 2.5 and 9.5 kHz to reduce background noise while maintaining full acoustic integrity across the song frequency range. These files were then segmented by syllable using RavenPro v1.5 by highlighting each syllable in a joint spectrogram and waveform view and compiling a “selection table” that includes start and end times for each selection. Syllables were defined as a single continuous sound separated from other sounds by a silent period longer than 0.015 s or as a group of sounds separated from each other by less than 0.015 s and separated from other sounds by more than 0.015 s. This definition was used to identify vocalizations most likely to be made during a single expiratory event; 0.015 s is a value that approximates the shortest recorded inspiratory event (“mini‐breath”) of any bird species (e.g., Goller & Daley, 2001; Hartley & Suthers, 1989). Two separate acoustic analyses were conducted: syllable morphology and rhythm.

The acoustic morphology of syllables was analyzed by taking automated measurements of each syllable with a custom R script that used dominant frequency tracking (dfreq function from seewave package; Sueur et al. 2008; R Core Team, 2017). The measurements used were as follows: mean dominant frequency, standard deviation of dominant frequency, minimum dominant frequency, 3rd quartile frequency, maximum dominant frequency, dominant frequency range, frequency modulation rate, centroid, mode, skewness, kurtosis, spectral flatness, and dominant frequency change index (see Appendix S1). These measurements were then included in a linear discriminant analysis (“lda” function in R). The lda was trained on the data from allopatric populations of Hermit and Townsend's warblers, and then applied to the full dataset, including samples from both allopatric (i.e., removed from the hybrid zone) and sympatric (i.e., within the hybrid zone) populations. As described in Results, syllable morphology LD1 scores reflect a complex set of acoustic features (see Figure S5 for song spectrogram showing LD1 scores for each syllable).

Rhythm was analyzed using custom code in R that assessed components of songs relating to the pattern of sound and silence (following procedures outlined in Love et al., 2019) without including frequency measures. Briefly, using the syllable start time and syllable duration from the RavenPro selection table, summary features of each song were computed in R. The rhythmic features that were recorded are as follows: mean syllable length (syllable end time minus syllable start time), mean silent period length (syllable end time minus start time of next syllable), syllable rate (number of syllables/song length), “syllable regularity,” and “silent period regularity.” Syllable regularity and silent period regularity are normalized measures of consistency. Syllable regularity was computed within a single song by first finding the proportion of total sound that each syllable represents (syllable length divided by the sum of all syllable lengths). Then, the proportions were normalized by dividing each proportion by the maximum proportion. These normalized proportions were summed, and the sum was divided by the total number of syllables in the song. The resulting value yielded the syllable regularity. The same procedure was utilized to produce the silent period regularity, substituting silent period lengths for syllable lengths. A linear discriminant function was then applied, following the same procedure that was used for syllable morphology. As described in Results, songs that receive more positive rhythm LD1 scores have fewer syllables, longer silent periods between syllables, and a lower syllable repetition rate with less consistent syllable duration. We quantified the degree of convergence in two ways: one as the proportion of sympatric birds predicted to be Hermit (for syllable morphology) or Townsend's (for rhythm) by linear discriminant classification, and one as the proportion of sympatric birds with LD1 scores that were negative (for syllable morphology) or positive (for rhythm).

To assess the statistical validity of our linear discriminant‐based findings, we conducted a randomization test. Briefly, we replicated our syllable morphology and rhythm data but randomly assigned each sample to a species identity. For each song module, we then trained 10,000 linear discriminant functions on simulated data, repeating above methods, and reported the two measures of convergence. We then determined the proportion of the distribution of simulated convergence statistics (here, the null distribution), which were equal to or more extreme than the values that we found with our real data. This proportion functions as a *p*‐value (Whitlock & Schluter, 2009).

It has been previously described that Hermit and Townsend's warblers have two main song types, which are “performance‐encoded” (Byers 1995) or distinguished by their apparent use (Spector 1992, Janes & Ryker, 2006, 2013, Janes and Ryker 2016). We conducted a thorough song‐type analysis and did not find strong evidence to support this distinction of songs (Love and Goller *unpublished*). We therefore include all song forms in the present study.

### 2.3 Geographic cline analyses

To visualize and quantify trait change across the hybrid zone, we conducted three one‐dimensional geographic cline analyses. The plumage‐based hybrid index scores and the first linear discriminant scores produced by the syllable morphology and rhythm linear discriminant functions served as trait data for the three analyses. Obtaining clines based on each of these traits allowed us to compare how each song trait evolves across the hybrid zone relative to the plumage trait, a reliable indicator of genetic background. If a song trait cline differs from the plumage cline in width, then we can infer that there is a difference between the strength of selection acting on plumage versus that acting on the song trait. If the clines differ in center location, then we can infer introgression between the two traits. Lastly, if the data cannot be effectively modeled by a cline, then it suggests that either parent species do not differ in this trait or that atypical patterns of inheritance are present.

We used the hzar package in R, following a protocol outlined in Derryberry et al. (2014) for quantitative traits and using default settings, no parameter constraints, and a single cycle of fit requests (two sequential model fittings per analysis) to reach a maximum‐likelihood cline, which gives a representation of the variation in a trait over a geographic area. Since these analyses relied on grouped sampling events, we binned our samples by every kilometer following a north–south axis. We used a direct north–south axis as our single geographic dimension, which fits the species and plumage score distribution across our study area and any other possible straight‐line transect (see Results) and is in line with past studies (Rohwer et al., 2001; Rohwer & Wood, 1998).

### 2.4 Playback experiment

We conducted a playback experiment in order to evaluate the social function of song traits in this system. By varying the rhythm and syllable morphology trait values of playback songs across trials and recording response magnitude of each individual, we intended to identify which features of song are socially salient in terms of module (rhythm versus syllable morphology) and trait value (Hermit‐like versus Townsend's‐like). By testing both males and females, we intended to determine the relative importance of song traits in functioning as a mating signal in male–female interactions or as an aggressive signal in male–male interactions.

The playback experiment was conducted in May–July 2016, 2017, and 2018. Locations were determined through a roving approach of the entire study area. Individuals were located by their song and then tracked to their perch. After an individual was located and its song recorded as above, playback was initiated. The playback song used was randomly selected from a suite of our previously recorded songs, which were selected for features described in the following text. Prior to use in the experiment, these songs were edited in the Praat software to remove any background noise with a band‐pass filter and spectral subtraction, using a window length of 0.025 s and a smoothing bandwidth of 1.0. After filtering, mean intensity of the song was scaled to 75 db. Peak intensity was not normalized in order to preserve any potentially informative differences between songs.

**FIGURE 2:**
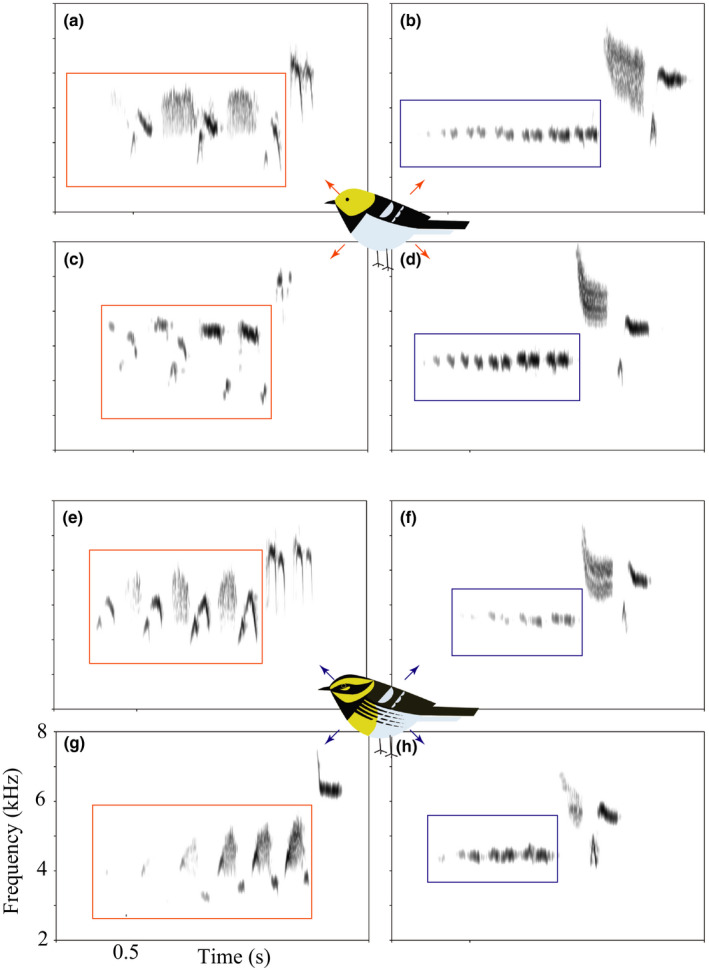
Playback songs of sympatric Hermit and Townsend's warblers varied by number of notes in the introductory syllables. Upper four (a–d) songs were sung by sympatric Hermit warblers, and lower four (e–h) songs were sung by sympatric Townsend's warblers. Allopatric Hermit warblers use multinote introductory syllables (red box, as in a, c, e, g), while allopatric Townsend's warblers use single‐note introductory syllables (blue box, as in b, d, f, h). In sympatry, both parent species use both multi‐ and single‐note intro syllables. Graphics are representative, showing divergent plumage

Preliminary analysis of song databases (xeno‐canto.org and Macaulay Library) showed a clear pattern in allopatric species‐specific song: Allopatric Townsend's warblers use single‐note introductory syllables, while allopatric Hermit warblers use multinote introductory syllables (see Appendix S1). With this in mind, we established a suite of 8 songs, each sung by a different bird, to use for playback that varied in introductory syllable morphology and species identity of the singer. 8 songs were used for playback (see Figure 2), 4 of which were produced by individuals visually scored as Townsend's warblers from the hybrid zone and 4 of which were produced by individuals from the hybrid zone visually scored as Hermit warblers. Of each set of 4, 2 had single‐note introductory syllables and 2 had multinote introductory syllables. So, the playback sample included songs sung by both species but which contained a primary characteristic of con‐ and heterospecific song (Figure 2). Songs to include in this suite needed to meet the qualifications above and be of high recording quality. Two control playback songs of locally common birds, one of a dark‐eyed junco (*Junco hyemalis*) and one of an American robin (*Turdus migratorius*), were included in the song suite to test for the possible effect of response by the focal species relying in part on a general disturbance response. At no time did the controls elicit a measurable or noticeable response from the target bird, so these controls are not included in the analyses.

To refine our design for the playback experiment, we conducted a pilot study (see Appendix S1). All trials included in our dataset used the following design. These species live in the forest canopy, so playback was initiated under the perched bird, immediately adjacent to the perch tree, in an exact spot selected for the availability of perches at continuously decreasing distances from the playback speaker, which was placed on the ground. Next to the speaker, we placed a clay mount, one side being painted with the plumage pattern of a Townsend's warbler, and the other side painted with the plumage pattern of a Hermit warbler, which was oriented so as to not favor one side over the other from the most readily available perches. 10‐m, 3‐m, and 1‐m distances from the speaker were measured with a premeasured lightweight pole, which was removed before playback began and marked by naturally available objects. With the experimenter standing over 10 m away in a location that allowed for observation of the full study area, playback was commenced. Songs were randomly selected for playback with a balanced design. A song was played at a rate of once every 3.5 s for 10 min, and magnitude of response to playback was continuously recorded. After the playback period was complete, photographs and additional song recordings were taken. Many individuals were tested a second time with a different playback song a minimum of 30 min after the end of the first playback period, an amount of time that was sufficient for the focal bird to resume normal behavior. Order of playback response was recorded (i.e., 1st or 2nd playback response) in the field and was subsequently included as a random effect in analyses.

Our final dataset includes a total of 215 playback trials with males (51 Hermit, 38 hybrid, and 124 Townsend's). Magnitude of response was defined using the continuously recorded response distance category (>10 m, <10 m, <3 m, <1 m) from the playback speaker and duration of time spent <1 m from the playback speaker. The summary statistic used to represent overall response in subsequent analyses was obtained by using the first principal component values after conducting a PCA (prcomp function in R) that included the nearest response distance (PC1 loading: −0.57), duration of time spent <1 m from the playback speaker (0.54), and the duration of time between the start of playback and the closest response (−0.62). PC1 explained 66% of the variance. For an additional direct comparison of male‐to‐female response, we scaled and centered the response data.

Since they are more difficult to detect and locate in the field, we could not reliably target females in the same way as males. Still, whenever we had direct confirmation that a female was in the immediate vicinity of the male‐directed playbacks described above, we recorded their behavior and plumage score (using the methods for males but calibrated for female plumage traits) as well. We included in our analyses only female responses, which were uninterrupted by a responding male. Females were occasionally observed showing interest in playback, but their potential approach was interrupted by the male who was the primary target of our playback. In this case, we observed that the responding male appeared to chase the female, sometimes aggressively, a behavior that suggested mate guarding by the male (Birkhead, 1979). This observation also suggests that response from the female to playback may indicate an intent to explore extra‐pair mating opportunities rather than to defend the territory. Sample sizes were much lower than for males (*n* = 32 total female responses recorded; 10 Hermit, 6 hybrid, and 16 Townsend's). Female responses were generally of a less aggressive nature than male responses. In contrast to our observations of males, we did not observe any female make contact with the playback speaker or mount, and their flights and behaviors appeared less determined. These observations again suggest that female response to playback did not reflect territory defense. We categorized female response more broadly as close (less than 3 m from the playback speaker), far (over 3 m away, but clearly altering their behavior by approaching the playback speaker), and ignore (did not clearly alter their behavior by approaching the playback speaker). For an additional direct comparison of male‐to‐female response, we scaled and centered the female close response data and combined them with the separately scaled male response data, then conducted an ANOVA with a sex‐by‐song rhythm interaction term.

In all analyses that used sympatry/allopatry status, we defined individuals as residing in sympatry when they were within 40 km of a heterospecific (or hybrid). Those that were more than 40 km from a heterospecific (or hybrid) were defined as residing in allopatry.

## 3 RESULTS

Hermit and Townsend's warbler males use different songs in allopatry but sing similar songs near the center of the hybrid zone. General patterns of trait change across the hybrid zone suggest overall convergence, with notable convergence on Townsend's‐like song rhythm, though the specifics of the patterns of change in the two song modules are different. The playback experiment revealed that females respond differently to song rhythm, while males show little difference in response to different song forms.

### 3.1 Acoustic analysis

Preliminary analysis of online databases of Hermit and Townsend's warbler songs yielded 141 Townsend's and 46 Hermit warbler songs. Visual inspection of these spectrograms showed a clear trend toward predominant use of multinote syllables by Hermit warblers, while Townsend's warblers use single‐note syllables in the first song phrase (see Appendix S1). This observation is consistent with the findings of Morrison and Hardy (1983). We used these results to frame the rest of our study.

**FIGURE 3:**
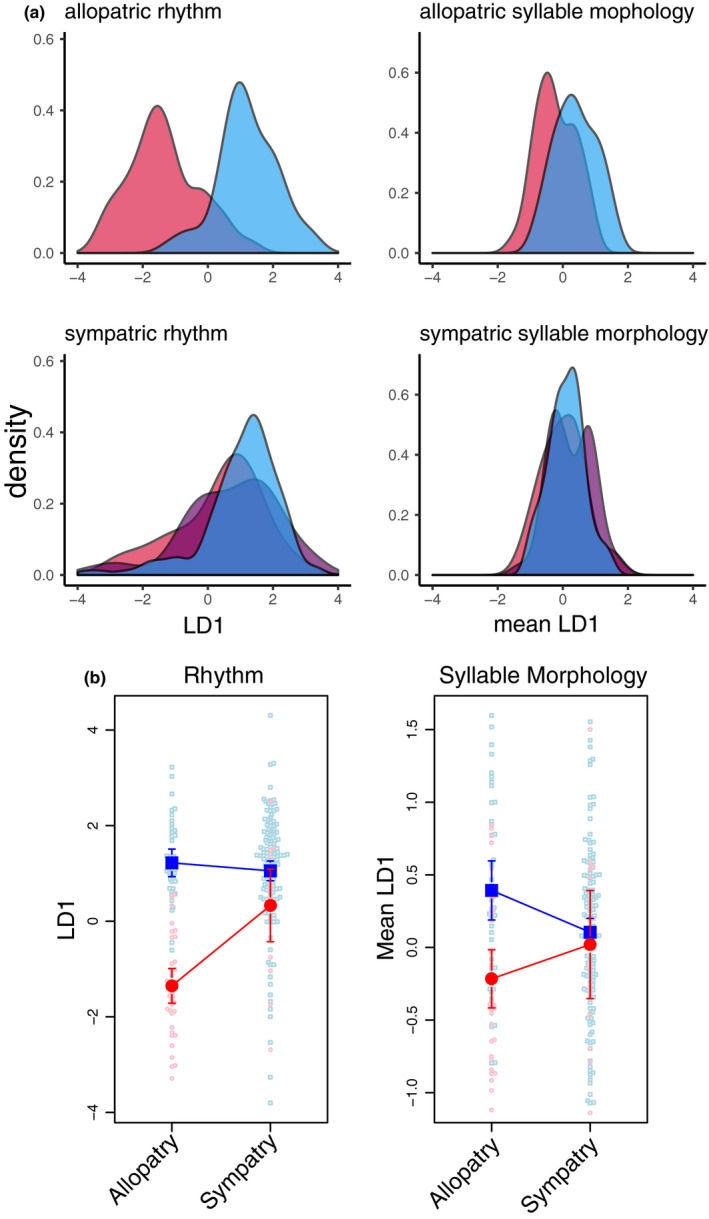
Asymmetrical convergence of song features in the hybrid zone. (a) Density distributions plots of Hermit (red), Townsend's (blue), and hybrid (purple) song features. Left panels: Largely distinct allopatric song rhythm converges on Townsend's‐like values (positive rhythm LD1) in sympatry. Right panels: Allopatric syllable morphology overlaps broadly, but means are significantly different. In sympatry, syllable morphology converges to intermediate values. (b) Hermit (red) and Townsend's (blue) song traits in allopatry and sympatry. Rhythm converges to Townsend's‐like values in sympatry, while syllable morphology converges to intermediate values in sympatry. Bold dots and bars show mean +95% CI

One part of our analysis of songs recorded in and immediately around the hybrid zone assessed song rhythm. Hermit warblers and Townsend's warblers use different song rhythms in allopatry, but song rhythm converges to Townsend's‐like values in areas of sympatry (Figure 3), suggesting a pattern of introgression. Coefficients of the first linear discriminant (LD1) in the linear discriminant analysis are shown in Table 1. Songs that receive more positive rhythm LD1 scores have fewer syllables, longer silent periods between syllables, and a lower syllable repetition rate with less consistent syllable duration. In allopatry, rhythm LD1 scores readily distinguished between Hermit and Townsend's warbler song. Townsend's warblers primarily sang songs with rhythm LD1 scores greater than zero (peak estimated at LD1 = 0.97), and Hermit warbler songs scored on rhythm LD1 below zero (peak estimated at LD1 = −1.56) (Figure 3a). The classification error rate of the rhythm linear discriminant analysis was 1%. In sympatry, rhythm scores for Hermit warbler song showed a positive shift (Figure 3b; red; peak at LD1 = 0.89). Townsend's warbler songs only showed a minor positive shift (Figure 3b; blue; peak at LD1 = 1.40). LD1 rhythm scores for hybrid songs were more broadly distributed with a positive LD1 peak (Figure 3b; violet; peak at LD1 = 1.41). The resulting pattern is that most songs from sympatric populations have rhythms that are similar to those produced in allopatric Townsend's warbler populations. This convergent pattern was assessed statistically using both the LDA‐predicted species category and the LD1 score. 84% of individuals in the sympatric population were predicted to be Townsend's based on LDA classification, and 81% of sympatric LD1 scores were positive.

**TABLE 1:** Coefficients of rhythm linear discriminant analysis

	LD1
Number of syllables	−1.09
Mean syllable duration	−0.18
Mean silent period duration	0.36
Song length	0.0052
Syllable rate	−0.4
Rhythmic silence regularity	0.032
Rhythmic sound regularity	−0.22

We compared these convergence statistics with the distribution of simulated statistics in randomization tests. Using the rhythm LDA classification‐based statistic, we found that 9,652/10,000 trials had a lesser degree of convergence than was found in the real data (*p* = .029). Using the rhythm LD1 score‐based convergence metric, an extreme majority of trials (9,926/10,000; *p*‐value = 0.0074) had a lower proportion of positive sympatric rhythm LD1 scores than was found in our dataset. In particular, since LD1 scores reflect acoustic traits more directly than LDA classification, the results of these tests together strongly suggest that the pattern of convergence in song rhythm to Townsend's‐like values is not the result of chance.

For the allopatric areas used in our study, Hermit warblers and Townsend's warblers showed broad overlap in syllable morphology and statistically significantly different mean trait values (song means of syllable scores of allopatric Hermit versus. Townsend's LD1; *t* = 4.31, *df* = 75.92, *p*‐value = 0.00005) (Figure 3a,b). In sympatry, syllable morphology converges to intermediate trait values (sympatric Hermit versus. Townsend's LD1; *t* = 0.47, *df* = 16.24, *p*‐value = 0.65) (Figure 3). Coefficients of the first linear discriminant (LD1) in the linear discriminant analysis are shown in Table 2. The classification error rate of the syllable morphology linear discriminant analysis was 34%.

**TABLE 2:** Coefficients of syllable morphology linear discriminant analysis

	LD1
Mean dominant freq	−0.039
Standard deviation dominant freq	−1.067
Max dominant freq	0.29
Min dominant freq	−0.55
75% dominant freq	−0.36
Dominant freq range	0.44
FM rate	−0.18
Centroid	0.21
Mode	−0.055
Skewness	0.44
Kurtosis	−0.39
Spectral flatness	0.47
Change index	−0.173

We quantified possible convergence to Hermit‐like syllable morphology as proportion of sympatric samples predicted by the syllable morphology LDA to be Hermits (75%) and as the proportion of syllables with negative LD1 scores (51%). Randomization tests using both the classification‐based statistic and the LD1‐based statistic suggest that, while syllable morphology may converge, it does not converge on Hermit‐like values more than expected by chance (classification: *p* = 1; LD1: *p* = .54).

### 3.2 Geographic cline analysis

**FIGURE 4:**
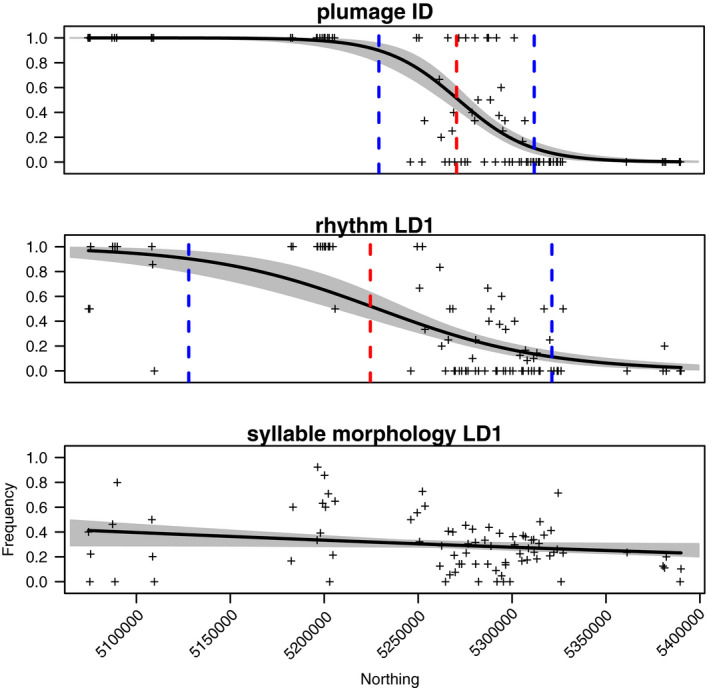
Geographic cline analysis. Samples were averaged every 1 km. Black line represents the maximum‐likelihood cline. Shaded region is 95% credible cline region. Red and blue lines indicate trait cline‐based center and edges of hybrid zone. Plots produced with HZAR package (Derryberry et al., 2014)

Song rhythm showed a clinal transition across the hybrid zone that is shifted to the south and less steep when compared to the plumage ID cline (Figure 4). In contrast, syllable morphology displayed negligible clinal change across the hybrid zone (Figure 4, bottom). One‐dimensional geographic cline analysis of plumage identity scores showed a cline with center at 5270800m N (UTM Zone 10N) and a width of 82.6 km. The cline created for rhythm LD1 scores had a center 46 km to the south at 5224637m N and a greater width of 193.4 km. A cline model did not fit syllable morphology LD1 scores well, and the cline center was defined well south of the study area (Figure 4, bottom).

### 3.3 Playback experiments

If individuals of these species use either introductory syllable morphology or song rhythm as a cue in territorial disputes or mate attraction, then we expected these tendencies to be expressed during song playback experiments. We found that categorical parameters (single/multiple introductory syllable morphology and species identity of the singer), around which we designed our playback, did not significantly affect response, but song rhythm LD1 scores did.

As described in Methods, composite response scores were generated by applying a PCA to raw response data. The composite response scores (PC1) show similar patterns to those of the raw data (see Appendix S1). A linear mixed‐effects model was constructed to assess the relationship between male response to song and the following explanatory variables: species identity of the responding bird, allopatry/sympatry status, introductory syllable morphology category of the playback song, and species identity of the playback song singer (“lmer,” lme4 package, *p*‐values from Satterthwaite's method). Only parent species male responses were considered in these analyses. Order of playback, Julian date, and stimulus song were included as random effects. Species identity of the responding bird showed a significant relationship (Townsend's warblers show greater response than Hermit warblers; *df* = 122.51, *t* = 2.46, *p* = .015), as did allopatry status (overall response is lower in sympatry than in allopatry; *df* = 123.81, *t* = 3.08, *p* = .0026). Interestingly, neither syllable morphology category nor species identity of the singer of the playback song had a significant effect on response (*df* = 5.19, *t* = −0.607, *p* = .57 and *df* = 5.40, *t* = 1.30, *p* = .25, respectively).

To further characterize these relationships, additional statistical analyses were conducted. Response to playback song was lower in the hybrid zone compared with that found in allopatric populations, and this trend appears to be heavily driven by a reduced response to song by Townsend's warblers in the sympatric population. When accounting for the additional random effects of allopatry status and both playback song‐associated variables, species identity showed a significant relationship with response such that Hermit warblers responded less overall than Townsend's warblers (lmer, *df* = 117.35, *t* = 2.34, *p* = .0021). When analyzed independently, Hermit warbler response did not appear to be impacted by allopatry or playback song variables (lmer, species ID: *df* = 43.88, *t* = 1.45, *p* = .15; syllable morphology: *df* = 40.62, *t* = 1.01, *p* = .32; allopatry: *df* = 44.27, *t* = 1.09, *p* = .28), while Townsend's warbler response showed a significant relationship with allopatry status (*df* = 50.57, *t* = 3.13, *p* = .0029). The response of the latter species displayed no significant effect of introductory syllable morphology (*df* = 105.49, *t* = −1.55, *p* = .048) or species identity of the playback song singer (*df* = 108.81, *t* = 1.72, *p* = .088).

Hybrid males did not respond significantly differently to Hermit versus Townsend's warbler song (lmer, hybrid data only with fixed effects for both playback song variables and order and Julian date as a random effect, *df* = 29.83, *t* = 0.078, *p* = .94) and, like the parent species males, showed no significant effect of syllable morphology on response (same lmer, *df* = 29.53, *t* = −1.03, *p* = .313).

Female response to song did not differ significantly by introductory syllable morphology category (pooled data for females of all species, Welch two‐sample *t* tests: *t* = −1.27, *df* = 29.97, *p* = .21) or by species identity of the playback singer (*t* = −1.74, *df* = 28.87, *p*‐value = 0.09). Sample sizes were too low to reliably compare female response in sympatric versus allopatric regions.

**FIGURE 5:**
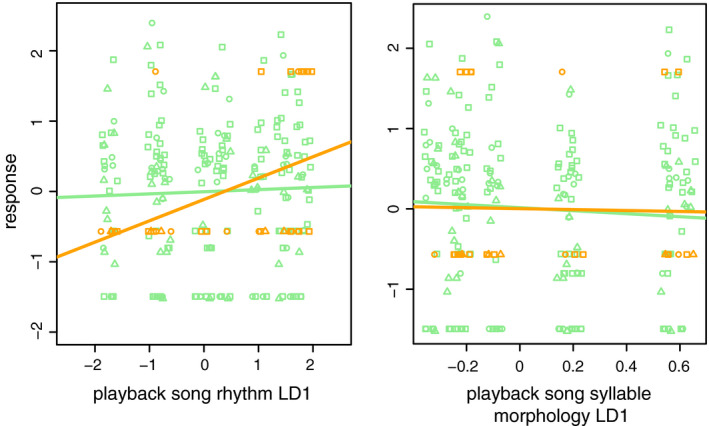
Variation in responses to different song rhythm (left) and average syllable morphology (right) LD1 scores by females (orange) and males (green). Circles are Hermits, squares are Townsend's, and triangles are hybrids. Lines show sex‐specific linear regressions. Female response is greater with positive (Townsend's‐like) rhythm LD1 scores (*p* = .017). Males and females show little difference in response to songs with different average syllable morphology. Points are jittered to increase visibility. Includes data from sympatric and allopatric populations

In addition to analyzing responses to song categories (single‐ vs. multinote syllables and species ID of singer), we also assessed the effect of average syllable morphology LD1 and rhythm LD1 scores on responses by males and females. We found that one of these features of song was more informative to response magnitude. Rhythm LD1 scores of playback songs ranged between −1.74 and 1.83 (−1.74, −0.91, −0.76, −0.011, 0.35, 1.00, 1.49, and 1.83). Males did not differ in response according to song rhythm (linear regression; *F* = 0.27, *df* = 211, adjusted *r*‐squared = −0.0035, *p* = .61), but females did (*F* = 6.39, *df* = 30, adjusted *r*‐squared = 0.15, *p* = .017) (Figure 5a). To confirm this sex difference in response, we conducted an ANOVA on response with a sex‐by‐rhythm LD1 interaction term (sex: *F* = 0, *p* = 1; rhythm: *F* = 2.08, *p* = .15; sex*rhythm: *F* = 3.723, *p* = .0548).

Playback songs ranged from −0.33 to 0.63 in average syllable morphology LD1 scores. Males and female response magnitudes did not show a strong relationship with mean syllable morphology LD1 scores (linear regressions; males: *t* = −0.95, *df* = 211, adjusted *r*‐squared = −0.00068, *p* = .36; females: *t* = −0.11, *df* = 30, adjusted *r*‐squared = 0.033, *p* = .91) (Figure 5b).

## 4 DISCUSSION

Our focus in this study of song in an avian hybrid zone was on the interactions among two primary sources of variation in birdsong (rhythm and syllable morphology) and two primary selective regimes predicted by theory to be active in such a system (selection for effective mate attraction and for efficient territory defense). We found that song traits change across the hybrid zone in a bipartite manner; song rhythm converges on Townsend's‐like values, while syllable morphology converges on intermediate values (Figures 3 and 4). The directions of this two‐part change may be explained by the result of a playback experiment: Females responded more strongly toward song with Townsend's‐like song rhythm (high rhythm LD1 scores), while the responses of neither sex appeared to differ based on syllable morphology (Figure 5). While uneven sample sizes limit our confidence to draw strong conclusions, we interpret this result to suggest that song rhythm is a main target of selection for effective mate attraction. If further study indicates that our interpretation is correct, rhythm and syllable morphology respond to different selective regimes, and song evolves in accordingly complex patterns.

Song rhythm and syllable morphology arise through two largely distinct song production modules. It is this independence, which allows the two aspects of one signal to evolve separately from one another. Song rhythm is produced through song‐associated respiratory patterning, which is thought to be largely innate in many species (Araki et al., 2016; Love et al., 2019; Marler & Sherman, 1983, 1985; Thorpe, 1958), while syllable morphology arises through syringeal motor gesturing, which is more likely to be learned to a greater extent (Ali et al., 2013; Love et al., 2019; Marler & Sherman, 1983, 1985). Irrespective of their different *directions* of change, the remarkably different *shapes* of change of rhythm and syllable morphology in our study (Figure 4) may reflect the respective inheritance modes of these two song production modules. Rhythm follows a clinal transition characteristic of vertical genetic inheritance (Mundinger, 1982, Barton and Hewitt, 1985, Isler et al. 2005, Nyári, 2007). In contrast, syllable morphology follows a nonclinal “mosaic” pattern, which is consistent with locally mediated cultural transmission in learned vocalizations (Podos & Warren, 2007), but which could potentially be the result of processes unrelated to vocal learning (Odom & Mennill, 2012). Further study is therefore warranted to confirm these findings. Learning of local song features is an important factor regulating song development (e.g., Akçay et al., 2013; Nordby et al., 2001), and so we suggest that cultural transmission of syllable morphology is a likely interpretation of the mosaic pattern of syllable morphology in this system.

Our suggestion that unlearned song rhythm plays a greater role in mate attraction than does learned syllable morphology is of great interest in the context of the evolution of vocal learning. Mate attraction has been suggested as a key contributor to the evolution of vocal learning, which in some species allows individuals to develop large repertoires that could aid in mate attraction (Catchpole and Slater, 2003). In particular, if confirmed by direct study of song development in these species, our results subtly suggest that unlearned song features could be more important than learned song features for mate attraction in this system. While studies in some other species find that learned song components are important in mate attraction (e.g., Hernandez et al., 2008; Spencer et al., 2003), further research into the role of unlearned song components in mate attraction for vocal learning species is warranted. Our observation that rhythm appears less likely to be learned than syllable morphology fits with data from other species (Love et al. 2019), providing a potential starting point for such future research.

We hypothesized that song modules would show separate and distinct patterns of change across the hybrid zone because of the interaction of selective pressures for effective territorial aggression and for effective mate attraction. While we found general support for our hypothesis, the specific patterns that we found were somewhat unexpected. Reinforcement theory predicts that in the case of reduced hybrid fitness, which exists in this system (Pearson, 2000; Pearson & Rohwer, 2000), mating signals should diverge. We did not find evidence of divergence between species for any aspect of song, and thus no evidence for reinforcement acting on song in this system. Pearson (2000) hypothesized that female preference for Townsend's warbler traits drives hybridization. Our results provide some support for this hypothesis. The apparent female tendency to respond more strongly to Townsend's‐like song rhythm may explain convergence of this feature to Townsend's‐like values, which could potentially act in opposition to reinforcing selection. In the absence of such a preference, we would expect reinforcement to drive divergence of song rhythm between species. However, both the overall pattern of convergence of song and the convergence in syllable morphology are consistent with convergent agonistic character displacement (Grether et al., 2017).

Past research has suggested that Townsend's males are more aggressive than Hermit males and that species differences in aggression could contribute to movement of this hybrid zone (Pearson, 2000; Pearson & Rohwer, 2000). Our results suggest that male Townsend's warblers showed higher response magnitude than male Hermit warblers, but that difference was only evident in allopatric populations. In sympatric regions, we did not find that response magnitude differed between the two species (Appendix S1, Figure S4).

To what degree variation in the reliance on learning of song modules differentially drives phenotypic change needs to be investigated further, and our suggested mechanisms present testable hypotheses. Indeed, studies of other avian hybrid zones find similar complex patterns of trait evolution characterized by a clinal convergent transition in song rhythm‐related features paired with a nonclinal pattern of syllable morphology‐related features (e.g., Lipshutz et al., 2017; Secondi et al., 2003). It may be the case that differences in reliance on learning between song production modules are observable in other birdsong hybrid zones. However, since many studies use a multidimensional scaling approach to song analysis, the distinction between learned and genetically determined song traits may go unnoticed or the patterns uncovered in the process of analysis may be difficult to interpret.

Given the theoretical predictions of reinforcement and convergent agonistic character displacement, our conclusion about selective forces in this system is well supported by our data. However, we cannot directly exclude other possible explanations for the observed patterns. Our data do not allow us to assess whether genetic covariance of song traits with other targets of selection or potential differences between species in their respective vocal learning program may also have played a role in the observed geographic patterns of song. In addition, our interpretation that female response to playback reflects mating attraction informs our view that selective forces for effective mate attraction act on song rhythm. If female response is motivated differently, such as by territory defense, then it suggests that females and males weigh their levels of territorial aggression based on different male song features and that selection for effective territory defense may act separately through males and females. The Townsend's‐Hermit warbler hybrid system would be an appropriate venue for further exploration of all of these possibilities, and the results of our study provide testable predictions.

Song is viewed as an important mating signal under strong sexual selection (Collins, 2004; Searcy & Nowicki, 2005). Song learning is expected to accelerate divergence of song between species. Rapid divergence of song between diverging species should, then, establish reproductive barriers quickly during the speciation process. Indeed, it has been hypothesized that the rapid diversification of vocal learning songbirds may be due to rapid evolution of song (Lachlan & Servedio, 2004). However, this hypothesis has been subject to continued discussion (Baptista & Trail, 1992; Olofsson et al., 2011; Seddon & Tobias, 2007; Slabbekoorn & Smith, 2002; Verzijden et al., 2012; Yeh, 2018, 2019). Overall, this study questions the notion that vocal learning accelerates speciation through rapid cultural changes in song. In the pair of recently diverged species in the current study, we find evidence that aspects of song that appear more innate may play a stronger role in mate attraction, and therefore, divergence in these aspects would be more likely to constitute a barrier to reproduction between species. It remains to be determined whether this observation applies to other taxa.

## CONFLICT OF INTEREST

None declared.

## AUTHOR CONTRIBUTIONS

**Jay Love:** Conceptualization (equal); data curation (equal); formal analysis (equal); funding acquisition (equal); investigation (equal); methodology (equal); project administration (equal); resources (equal); software (equal); supervision (equal); validation (equal); visualization (equal); writing–original draft (lead); writing–review and editing (lead). **Franz Goller:** Conceptualization (equal); data curation (equal); formal analysis (equal); funding acquisition (equal); investigation (equal); methodology (equal); project administration (equal); resources (equal); software (equal); supervision (lead); validation (equal); visualization (equal); writing–original draft (supporting); writing–review and editing (supporting).

## Supporting information

Appendix S1

Appendix S2

## Data Availability

Data supporting our results are included in the supplement and on Dryad at https://doi.org/10.5061/dryad.n5tb2rbvk. Additional supporting data are available upon request.

## References

[ece37559-bib-0001] Akçay, Ç. , Tom, M. E. , Campbell, S. E. , & Beecher, M. D. (2013). Song type matching is an honest early threat signal in a hierarchical animal communication system. Proceedings of the Royal Society B, 280, 20122517. 23378665 10.1098/rspb.2012.2517 PMC3574360

[ece37559-bib-0002] Ali, F. , Otchy, T. M. , Pehlevan, C. , Fantana, A. L. , Burak, Y. , & Ölveczky, B. P. (2013). The basal ganglia is necessary for learning spectral, but not temporal, features of birdsong. Neuron, 80(2), 494–506. 10.1016/j.neuron.2013.07.049 24075977 PMC3929499

[ece37559-bib-0003] Araki, M. , Bandi, M. M. , & Yazaki‐Sugiyama, Y. (2016). Mind the gap: Neural coding of species identity in birdsong prosody. Science, 354(6317), 1282–1287. 10.1126/science.aah6799 27940872

[ece37559-bib-0004] Baptista, L. F. , & Trail, P. W. (1992). The role of song in the evolution of passerine diversity. Systematic Biology, 41, 242–247. 10.1093/sysbio/41.2.242

[ece37559-bib-0006] Barton, N. H. , & Hewitt, G. M. (1985). Analysis of hybrid zones. Annual Review of Ecology and Systematics, 16, 113–148. 10.1146/annurev.es.16.110185.000553

[ece37559-bib-0009] Birkhead, T. R. (1979). Mate guarding in the magpie Pica pica. Animal Behavior, 27, 866–874. 10.1016/0003-3472(79)90024-1

[ece37559-bib-0010] Brelsford, A. , Toews, D. P. L. , & Irwin, D. E. (2017). Admixture mapping in a hybrid zone reveals loci associated with avian feather coloration. Proceedings of the Royal Society B, 284, 20171106. 10.1098/RSPB.2017.1106 29118129 PMC5698634

[ece37559-bib-0092] Byers, B. E. (1995). Song types, repertoires and song variability in a population of chestnut‐sided warblers. The Condor, 97(2), 390–401. 10.2307/1369025

[ece37559-bib-0012] Catchpole, C. K. , & Slater, P. J. (2003). Bird song: Biological themes and variations. Cambridge University Press.

[ece37559-bib-0013] Cody, L. (1969). Convergent characteristics in sympatric species: A possible relation to interspecific competition and aggression. Condor, 71, 222–239. 10.2307/1366300

[ece37559-bib-0014] Collins, S. (2004). Vocal fighting and flirting: The functions of birdsong. In P. Marler & H. Slabbekoorn (Ed.), Nature's Music: The Science of Birdsong (pp. 39–79). Elsevier.

[ece37559-bib-0015] Coyne, J. A. , & Orr, H. A. (2004). Speciation. Sinauer Associates Inc.

[ece37559-bib-0018] de Kort, S. R. , den Hartog, P. M. , & ten Cate, C. (2002). Diverge or merge? The effect of sympatric occurrence on the territorial vocalizations of the vinaceous dove *Streptopelia vinacea* and the ring‐necked dove *S. capicola* . Journal of Avian Biology, 33(2), 150–158. 10.1034/j.1600-048X.2002.330205.x

[ece37559-bib-0019] Derryberry, E. P. , Derryberry, G. E. , Maley, J. M. , & Brumfield, R. T. (2014). Hzar: Hybrid zone analysis using an R software package. Molecular Ecology Resources, 14(3), 652–663. 10.1111/1755-0998.12209 24373504

[ece37559-bib-0020] Endler, J. A. (1992). Signals, signal conditions, and the direction of evolution. American Naturalist, 139, S125–S153. 10.1086/285308

[ece37559-bib-0022] Goller, F. , & Daley, M. A. (2001). Novel motor gestures for phonation during inspiration enhance the acoustic complexity of birdsong. Proceedings of the Royal Society B‐Biological Sciences, 268(1483), 2301–2305. 10.1098/rspb.2001.1805 PMC1088880 11703869

[ece37559-bib-0023] Grant, B. , & Grant, P. (1996). Cultural inheritance of song and its role in the evolution of Darwin’s finches. Evolution, 50(6), 2471–2478. 10.1111/j.1558-5646.1996.tb03633.x 28565664

[ece37559-bib-0024] Grether, G. F. , Peiman, K. S. , Tobias, J. A. , & Robinson, B. W. (2017). Causes and consequences of behavioral interference between species. Trends in Ecology & Evolution, 32(10), 760–772. 10.1016/j.tree.2017.07.004 28797610

[ece37559-bib-0025] Haavie, J. , Borge, T. , Bures, S. , Garamszegi, L. Z. , Lampe, H. M. , Moreno, J. , Qvarnström, A. , Török, J. , & Sætre, G. P. (2004). Flycatcher song in allopatry and sympatry ‐ Convergence, divergence and reinforcement. Journal of Evolutionary Biology, 17(2), 227–237. 10.1111/j.1420-9101.2003.00682.x 15009256

[ece37559-bib-0026] Halfwerk, W. , Dingle, C. , Brinkhuizen, D. M. , Poelstra, J. W. , Komdeur, J. , & Slabbekoorn, H. (2016). Sharp acoustic boundaries across an altitudinal avian hybrid zone despite asymmetric introgression. Journal of Evolutionary Biology, 29(7), 1356–1367. 10.1111/jeb.12876 27037611

[ece37559-bib-0027] Hartley, R. S. , & Suthers, R. A. (1989). Airflow and pressure during canary song: Direct evidence for mini‐breaths. Journal of Comparative Physiology A, 165(1), 15–26. 10.1007/BF00613795

[ece37559-bib-0029] Hernandez, A. M. , Phillmore, L. S. , & MacDougall‐Shackleton, S. A. (2008). Effects of learning on song preferences and Zenk expression in female songbirds. Behavioral Processes, 27(2), 278–284. 10.1016/j.beproc.2007.11.001 18155363

[ece37559-bib-0031] Irwin, D. E. , Thimgan, M. P. , & Irwin, J. H. (2008). Call divergence is correlated with geographic and genetic distance in greenish warblers (*Phylloscopus trochiloides*): A strong role for stochasticity in signal evolution? Journal of Evolutionary Biology, 21(2), 435–448. 10.1111/j.1420-9101.2007.01499.x 18205774

[ece37559-bib-0032] Isler, M. L. , Isler, P. R. , & Brumfield, R. T. (2005). Clinal variation in vocalizations of an antbird (Thamnophilidae) and implications for defining species limits. The Auk, 122, 433–444. 10.1642/0004-8038

[ece37559-bib-0033] Janes, S. W. , & Ryker, L. (2006). Singing of hermit warblers: Dialects of type I songs. Condor, 108(2), 336–347.

[ece37559-bib-0034] Janes, S. W. , & Ryker, L. (2013). Rapid change in a type I song dialect of hermit warblers (*Setophaga occidentalis*). The Auk, 130(1), 30–35. 10.1525/auk.2012.11273

[ece37559-bib-0093] Janes, S. W. , & Ryker, L. (2016). Type I and II songs of Townsend's warblers in Oregon and Washington. Western Birds, 47, 67–73.

[ece37559-bib-0035] Kenyon, H. L. , Toews, D. P. L. , & Irwin, D. E. (2011). Can song discriminate between MacGillivray’s and mourning warblers in a narrow hybrid zone? Condor, 113, 655–663. 10.1525/cond.2011.100182

[ece37559-bib-0036] Kirschel, A. N. G. , Blumstein, D. T. , & Smith, T. B. (2009). Character displacement of song and morphology in African tinkerbirds. Proceedings of the National Academy of Sciences of the United States of America, 106(20), 8256–8266. 10.1073/pnas.0810124106 19420223 PMC2688847

[ece37559-bib-0037] Krosby, M. , & Rohwer, S. (2010). Ongoing movement of the hermit warbler X Townsend’s warbler hybrid zone. Fenton B, editor. PLoS One, 5(11), e14164. 10.1371/journal.pone.0014164 21152406 PMC2994780

[ece37559-bib-0038] Lachlan, R. F. , & Servedio, M. R. (2004). Song learning accelerates allopatric speciation. Evolution, 58, 2049–2063. 10.1111/j.0014-3820.2004.tb00489.x 15521461

[ece37559-bib-0039] Laiolo, P. (2012). Interspecific interactions drive cultural co‐evolution and acoustic convergence in syntopic species. Journal of Animal Ecology, 81(3), 594–604. 10.1111/j.1365-2656.2011.01946.x 22260650

[ece37559-bib-0040] Lipkind, D. , Zai, A. T. , Hanuschkin, A. , Marcus, G. F. , Tchernichovski, O. , & Hahnloser, R. H. R. (2017). Songbirds work around computational complexity by learning song vocabulary independently of sequence. Nature Communications, 8(1), 1–11. 10.1038/s41467-017-01436-0 PMC5663719 29089517

[ece37559-bib-0041] Lipshutz, S. E. , Overcast, I. A. , Hickerson, M. J. , Brumfield, R. T. , & Derryberry, E. P. (2017). Behavioural response to song and genetic divergence in two subspecies of white‐crowned sparrows (*Zonotrichia leucophrys*). Molecular Ecology, 26(11), 3011–3027. 10.1111/mec.14002 28036146

[ece37559-bib-0042] Love, J. , Hoepfner, A. , & Goller, F. (2019). Song feature specific analysis of isolate song reveals interspecific variation in learned components. Developmental Neurobiology, 79(4), 350–369. 10.1002/dneu.22682 31002477

[ece37559-bib-0043] Lovette, I. J. , & Bermingham, E. (1999). Explosive speciation in the New World Dendroica warblers. Proceedings of the Royal Society B, 266, 1629–1636. 10.1098/rspb.1999.0825

[ece37559-bib-0045] Marler, P. (1960). Bird songs and mate selection. In W. E. Lanyon , & W. N. Tavolga (Eds.), Animal sounds and communication (Vol. 7, pp. 348–367). AIBS.

[ece37559-bib-0046] Marler, P. (1970). A comparative approach to vocal learning: Song development in white‐crowned sparrows. Journal of Comparative and Physiological Psychology, 71(2), 1–25. 10.1037/h0029144

[ece37559-bib-0048] Marler, P. , & Sherman, V. (1983). Song structure without auditory feedback: Emendations of the auditory template hypothesis. Journal of Neuroscience, 3(3), 517–531. 10.1523/JNEUROSCI.03-03-00517.1983 6827307 PMC6564553

[ece37559-bib-0049] Marler, P. , & Sherman, V. (1985). Innate differences in singing behavior of sparrows reared in isolation from adult conspecific song. Animal Behavior, 33(1), 57–71.

[ece37559-bib-0050] Morrison, M. L. , & Hardy, J. W. (1983). Hybridization between hermit and Townsend’s warblers. The Murrelet, 64, 65–72. 10.2307/3535264

[ece37559-bib-0051] Morton, E. S. (1975). Ecological sources of selection on avian sounds. American Naturalist, 109(965), 17–34. 10.1086/282971

[ece37559-bib-0052] Mundinger, P. C. (1982). Microgeographic and macrogeographic variation in the acquired vocalizations of birds. In D. E. Kroodsma , E. H. Miller , & H. Quellet (Eds.), Acoustic Communication in Birds (pp. 147–208). Academic Press.

[ece37559-bib-0056] Nordby, J. C. , Campbell, S. E. , & Beecher, M. D. (2001). Late song learning in song sparrows. Animal Behaviour, 61, 835–846. 10.1006/anbe.2000.1673

[ece37559-bib-0058] Nyári, Á. S. (2007). Phylogeographic patterns, molecular and vocal differentiation, and species limits in *Schiffornis turdina* (Aves). Molecular Phylogenetics and Evolution, 44, 154–164. 10.1016/j.ympev.2007.02.020 17412614

[ece37559-bib-0059] Odom, K. J. , & Mennill, D. J. (2012). Inconsistent geographic variation in the calls and duets of Barred Owls (*Strix varia*) across an area of genetic introgression. The Auk, 129, 387–398. 10.1525/auk.2012.11210

[ece37559-bib-0060] Okamoto, K. W. , & Grether, G. F. (2013). The evolution of species recognition in competitive and mating contexts: The relative efficacy of alternative mechanisms of character displacement. Ecology Letters, 16(5), 670–678. 10.1111/ele.12100 23489334

[ece37559-bib-0061] Olofsson, H. , Frame, A. M. , & Servedio, M. R. (2011). Can reinforcement occur with a learned trait? Evolution, 65, 1992–2003. 10.1111/j.1558-5646.2011.01286.x 21729054

[ece37559-bib-0062] Pearson, S. (1997). Behavioral and ecological tests of four models explaining narrow hybrid zones between hermit and Townsend’s warblers. (PhD dissertation). University of Washington.

[ece37559-bib-0063] Pearson, S. (2000). Behavioral asymmetries in a moving hybrid zone. Behavioral Ecology, 11, 84–92. 10.1093/beheco/11.1.84

[ece37559-bib-0064] Pearson, S. , & Rohwer, S. (1998). Influence of breeding phenology and clutch size on hybridization between hermit and Townsend’s warblers. The Auk, 115(3), 739–745. 10.2307/4089421

[ece37559-bib-0065] Pearson, S. , & Rohwer, S. (2000). Asymmetries in male aggression across an avian hybrid zone. Behavioral Ecology, 11, 93–101. 10.1093/beheco/11.1.93

[ece37559-bib-0066] Pfennig, K. S. , & Pfennig, D. W. (2009). Character displacement: Ecological and reproductive responses to a common evolutionary problem. The Quarterly Review of Biology, 84(3), 253–276. 10.1086/605079 19764283 PMC3279117

[ece37559-bib-0067] Podos, J. , & Warren, P. S. (2007). The evolution of geographic variation in birdsong. Advances in the Study of Behaviour, 37, 403–458. 10.1016/S0065-3454(07)37009-5

[ece37559-bib-0068] R Core Team (2017). R: A language and environment for statistical computing.

[ece37559-bib-0069] Rohwer, S. , Bermingham, E. , & Wood, C. (2001). Plumage and mitochondrial DNA haplotype variation across amoving hybrid zone. Evolution, 55(2), 405–422. 10.1111/j.0014-3820.2001.tb01303.x 11308096

[ece37559-bib-0070] Rohwer, S. , & Wood, C. (1998). Three hybrid zones between hermit and Townsend’s warblers in Washington and Oregon. The Auk, 115, 284–310. 10.2307/4089188

[ece37559-bib-0071] Searcy, W. A. , & Nowicki, S. (2005). The evolution of animal communication: Reliability and deception in signaling systems. Princeton University Press.

[ece37559-bib-0072] Secondi, J. , Bretagnolle, V. , Compagnon, C. , & Faivre, B. (2003). Species‐specific song convergence in a moving hybrid zone between two passerines. Biological Journal of the Linnean Society, 80, 507–517. 10.1046/j.1095-8312.2003.00248.x

[ece37559-bib-0073] Seddon, N. , & Tobias, J. A. (2007). Song divergence at the edge of Amazonia: An empirical test of the peripatric speciation model. Biological Journal of the Linnean Society, 90(1), 173–188. 10.1111/j.1095-8312.2007.00753.x

[ece37559-bib-0075] Slabbekoorn, H. , & Smith, T. (2002). Bird song, ecology and speciation. Philosophical Transactions of the Royal Society of London. Series B, Biological Sciences, 357, 493–503. 10.1098/rstb.2001.1056 12028787 PMC1692962

[ece37559-bib-0094] Spector, D. (1992). Wood‐Warbler song systems. Current Ornithology, 9, 199–238.

[ece37559-bib-0076] Spencer, K. A. , Buchanan, K. L. , Goldsmith, A. R. , & Catchpole, C. K. (2003). Song as an honest signal of developmental stress in the zebra finch (Taeniopygia guttata). Hormones and Behavior, 44(2), 132–139. 13129485 10.1016/s0018-506x(03)00124-7

[ece37559-bib-0077] Sueur, J. , Aubin, T. , & Simonis, C. (2008). seewave: A free modular tool for sound analysis and synthesis. Bioacoustics, 18, 213–226. 10.1080/09524622.2008.9753600

[ece37559-bib-0080] Thorpe, W. H. (1958). The learning of song patterns by birds, with especial reference to the song of the chaffinch *Fringilla coelebs* . Ibis, 100(4), 535–570. 10.1111/j.1474-919X.1958.tb07960.x

[ece37559-bib-0082] Tobias, J. A. , & Seddon, N. (2009). Signal design and perception in *Hypocnemis* antbirds: Evidence for convergent evolution via social selection. Evolution, 63(12), 3168–3189. 10.1111/j.1558-5646.2009.00795.x 19659594

[ece37559-bib-0083] Verzijden, M. N. , ten Cate, C. , Servedio, M. R. , Kozak, G. M. , Boughman, J. W. , & Svensson, E. I. (2012). The impact of learning on sexual selection and speciation. Trends in Ecology & Evolution, 27(9), 511–519. 10.1016/j.tree.2012.05.007 22705159

[ece37559-bib-0084] Wang, S. , Rohwer, S. , de Zwaan, D. R. , Toews, D. P. L. , Lovette, I. J. , Mackenzie, J. , & Irwin, D. (2019). Selection on a pleiotropic color gene block underpins early differentiation between two warbler species. bioRxiv. 10.1101/853390. Preprint PMC7719548 33312686

[ece37559-bib-0085] Wang, S. , Rohwer, S. , Delmore, K. , & Irwin, D. E. (2019). Cross‐decades stability of an avian hybrid zone. Journal of Evolutionary Biology, 32, 1242–1251. 10.1111/jeb.12524 31430391

[ece37559-bib-0086] West‐Eberhard, M. J. (1983). Sexual selection, social competition, and speciation. The Quarterly Review of Biology, 58(2), 155–183. 10.1086/413215

[ece37559-bib-0087] Whitlock, M. C. , & Schluter, D. (2009). The analysis of biological data. Roberts and Company Publishers.

[ece37559-bib-0088] Wilkins, M. R. , Seddon, N. , & Safran, R. J. (2013). Evolutionary divergence in acoustic signals: Causes and consequences. Trends in Ecology & Evolution, 28(3), 156–166. 10.1016/j.tree.2012.10.002 23141110

[ece37559-bib-0089] Yeh, D. J. (2018). The interaction between learning and speciation. PhD thesis. University of North Carolina.

[ece37559-bib-0090] Yeh, D. J. (2019). Assortative mating by an obliquely transmitted local cultural trait promotes genetic divergence: A model. American Naturalist, 193(1), 81–92. 10.1086/700958 30624103

