# Supplementary material for: Processes underlying complex patterns of song trait evolution in a Setophaga hybrid zone
Source: Ecol Evol. 2021 May 1;11(12):7264–77. doi: 10.1002/ece3.7559 (PMC8216987; doi:10.1002/ece3.7559)
Supplement: Supplementary file 1 — Appendix S1 [file ECE3-11-7264-s002.docx]

Appendix 1

Broad-level preliminary acoustic analysis

We performed a preliminary analysis of Hermit and Townsend’s warbler songs from publicly accessible databases, and we used the findings to frame much of our study. We noticed in a previous description of Hermit and Townsend’s warbler song (Morrison and Hardy 1983) that it appeared that the two species had clearly divergent introductory syllable morphology. To see if this was the case, we compiled recordings of both parent species from xeno-canto.org and the Cornell University Lab of Ornithology Macaulay Library (see appendix file). All songs from these databases that met standards of time of year (after spring and before fall migration: defined as May 15^th^ to August 8^th^), acoustic quality, and validity were included, resulting in a sample of 141 Townsend’s warbler and 46 Hermit warbler songs. JL visually inspected spectrograms of each song and categorized them as either “multiple-note introductory syllable” or as “single note introductory syllable” songs. We briefly report the results of our qualitative categorical song study here to provide context for the results of our more detailed analyses and playback experiment. Through observation of the dataset, a qualitative distinction between species-specific songs in allopatric populations became readily apparent: allopatric Townsend’s warblers use songs with single-note introductory syllables, while allopatric Hermit warblers use songs with multiple-note introductory syllables (see Figure 1 for examples). This characterization fits with the findings of previous literature on the subject (Morrison and Hardy 1983, Janes and Ryker 2006, 2016).

Of the 43 Townsend’s warbler samples from the Macaulay Library, 2 songs, or 5% of the total, from allopatric populations used multi-note introductory syllables: one near Glacier Bay in Alaska and one on Vancouver Island, British Columbia. There were 5 other samples in the Macaulay Library that used multi-note introductory syllables, but these samples did not meet standards for inclusion. Additionally, of the 98 Townsend’s warbler song samples in the online citizen-science nature sound repository xeno-canto.org, 21 used multi-note introductory syllables. Of those 21, 14 were from Vancouver Island, immediately to the north of the Olympic hybrid zone, 5 were from Gwaii Haanas Island in extreme western British Columbia, 1 was from Montana, and 1 was from Central B.C. Multi-note introductory syllables are not common in allopatric populations of Townsend’s warblers that are removed from hybrid zones. Overall, 4% of published recordings of the inland populations (East of the Coast Range) and ~45% of recordings from coastal populations (West of the Coast Range) of Townsend’s warblers use multi-note introductory syllables. The bulk of the coastal multi-note syllables are from Vancouver Island, again, immediately to the North of the Olympic hybrid zone. It is possible that this pattern results from dispersal from the hybrid zone to Vancouver Island.

Hermit warblers also appear to show an interesting trend in the morphology of their introductory syllables. In an allopatric population of Hermit warblers in northern Oregon, all encountered individuals sang songs with multi-note introductory syllables (n=12 individuals recorded by JL in 2016). Out of 46 recordings of Hermit Warbler song from the Macaulay library, 42 (91.3%) use multi-note introductory syllables. The 4 songs (8.7%) that use single-note introductory syllables are from the vicinity of Mount Hood in Oregon, the location of another hybrid zone (Rohwer and Wood 1998).

Confirming that syllable morphology LD1 captures known species-specific song features

To ensure that LDA dimensionality-reduction does not obscure relevant song features, we compared syllable morphology LD1 as defined in the methods to categorical introductory syllable morphology as described above for a subset of songs. Syllable morphology LD1 is substantially higher in songs that are characterized as having single-note introductory syllables than in songs that have multi-note introductory syllables (Welch two-sample t-test; t=-8.6, df=178.5, p-value=<0.00001; Figure S1). While the possibility remains that our techniques miss some unknown socially important song feature, it does appear to capture features of song that are
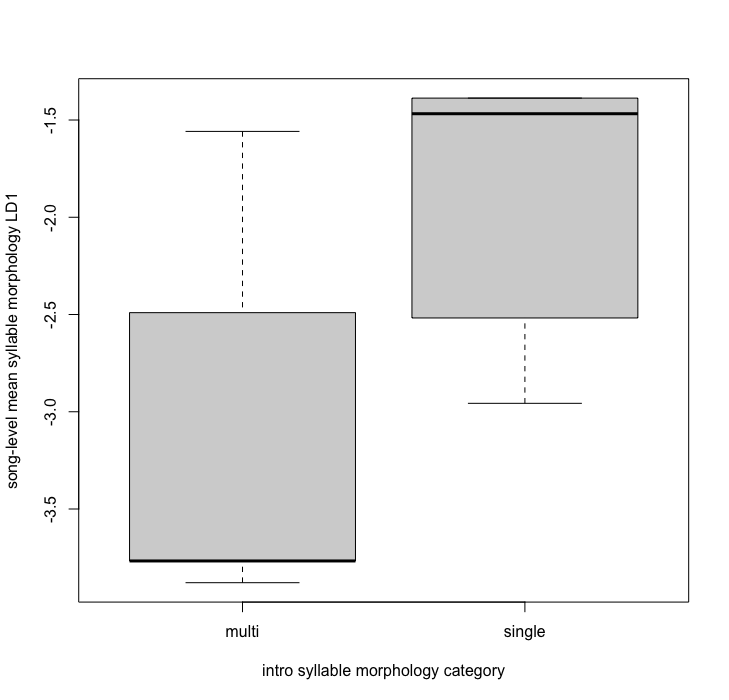
known to be divergent between the two species.

Figure S1. Syllable morphology LD1 effectively characterizes known syllable types.

Playback pilot

In order to refine our playback experiment, we performed a pilot study. The data from this pilot was not included in the playback response analyses. In early trials where we did not use a clay mount, playback elicited responses, but the responding bird often flew around the forest floor, searching a wide area for the intruder, making it difficult to quantify the response accurately. So, we created a clay mount to use, but did not want to bias response based on plumage patterning of Hermit vs. Townsend’s waarblers, so we painted one side of the mount with Townsend’s plumage and the other side with Hermit plumage. We then conducted a trial wherein the same individuals were tested twice, once with each half of the mount covered. This test did not reveal a clear effect of mount type on the response measures used in this study. In both the pilot and the full experiment, care was taken to avoid placing the mount so that the most prevalent perches would favor display of one side over the other. The use of a mount focused the responding bird’s attention to one point (the mount was even physically attacked several times) but avoided the potential of a specific coloration, rather than the song features, affecting the response. In order to specifically address our question of the role of song in eliciting aggressive interactions, we did not include measures of direct physical aggression in our response PCA (i.e., no pecks, hits, or wing flicks), which are behaviors likely to be more strongly induced by close-range signals such as plumage coloration than by comparatively long-range signals such as song. In addition, we determined through trial-and-error that a playback repetition rate of 1/3.5s and a playback duration of 10 minutes consistently produced responses sufficient for measurement. These values are within the range of values recorded by singing birds in the field.

Addressing the potential effects of body size on syllable morphology

Fundamental frequency tracks body size quite well (explains ~40% of the variation of a large survey across many species). Though there is a high degree of overlap in body size between the two parent species, Hermits are, on average, slightly larger than Townsend’s warblers. However, the data suggest broad overlap of frequency use, with Hermits perhaps using higher frequencies than Townsend’s do, opposite of the expected trend if frequency tracked body size closely. For these reasons, we are confident that the syllable morphology LD1 does not represent body size. See figure S1, below.


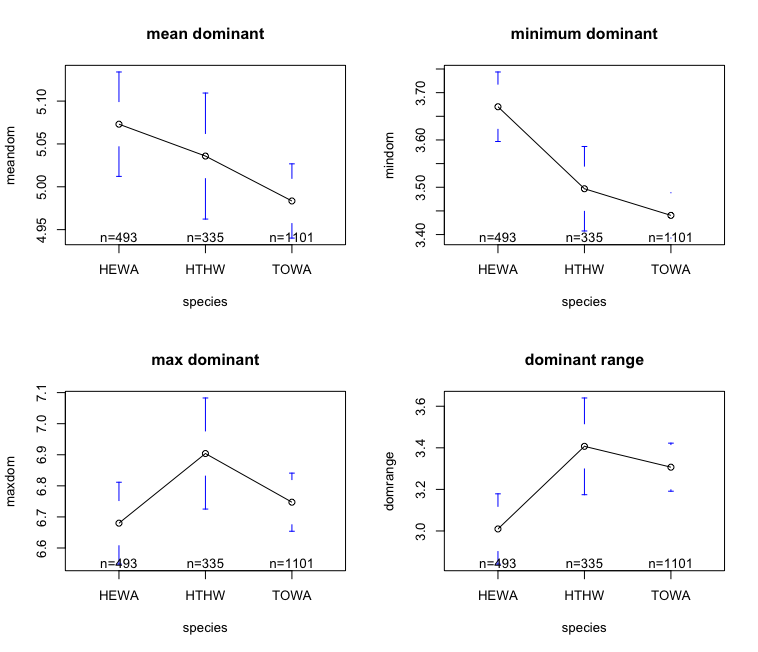


Figure S2: Comparisons of frequency measures across groups. HEWA = Hermit warbler, HTHW = hybrid, TOWA = Townsend’s warbler. Mean + 95%CI.

Seasonal effects of female response

Seasonal timing also affected female response: response was higher later in the season (multiple regression; rhythm LD1: t=2.29, df=30, P=0.030; Julian day: t=3.12, P=0.0040), though it should be noted that no females were detected after day 168 (approx. June 16^th^). However, the low sample size of female responses requires cautious interpretation; local singing or social conditions may play a role in female response (Figure S2).


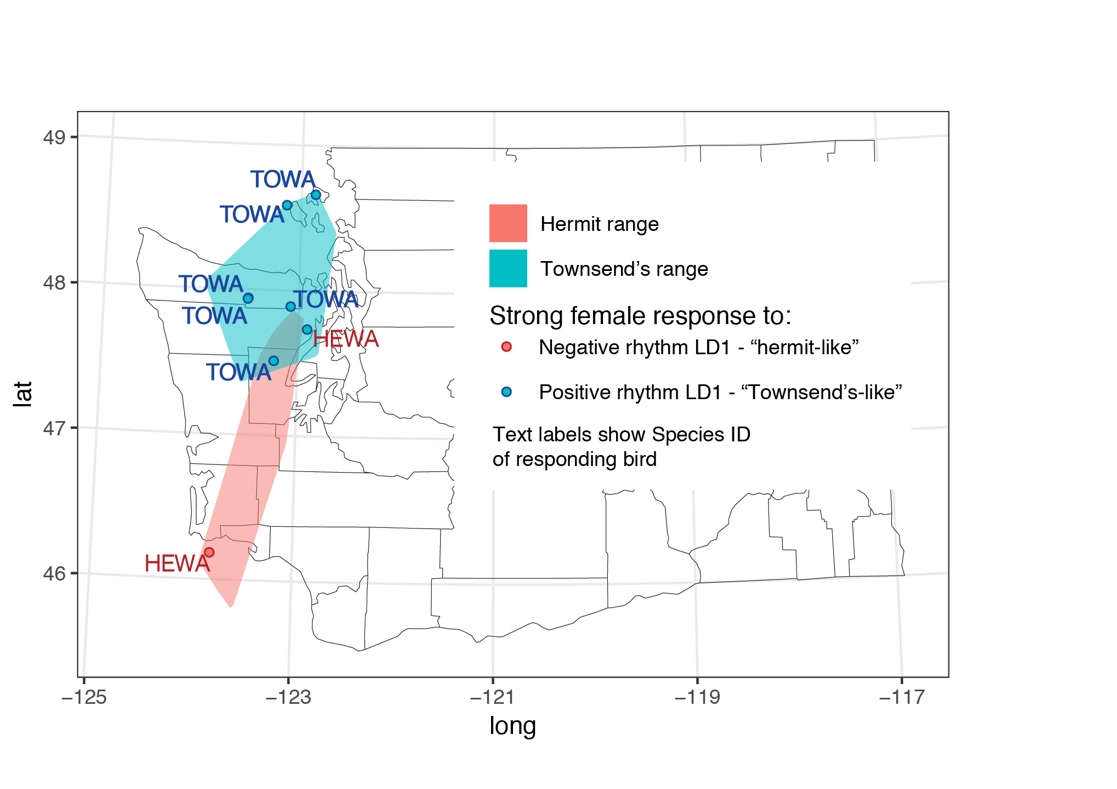


Figure S3: Map of female strong responses to playback. Most strong female responses to song are female Townsend’s warblers responding to “Townsend’s-like” song rhythm in both allopatry and sympatry. Two strong responses were from female Hermit warblers: one in allopatry which responded strongly to Hermit-like song rhythm, and one in sympatry which responded to Townsend’s-like song rhythm.


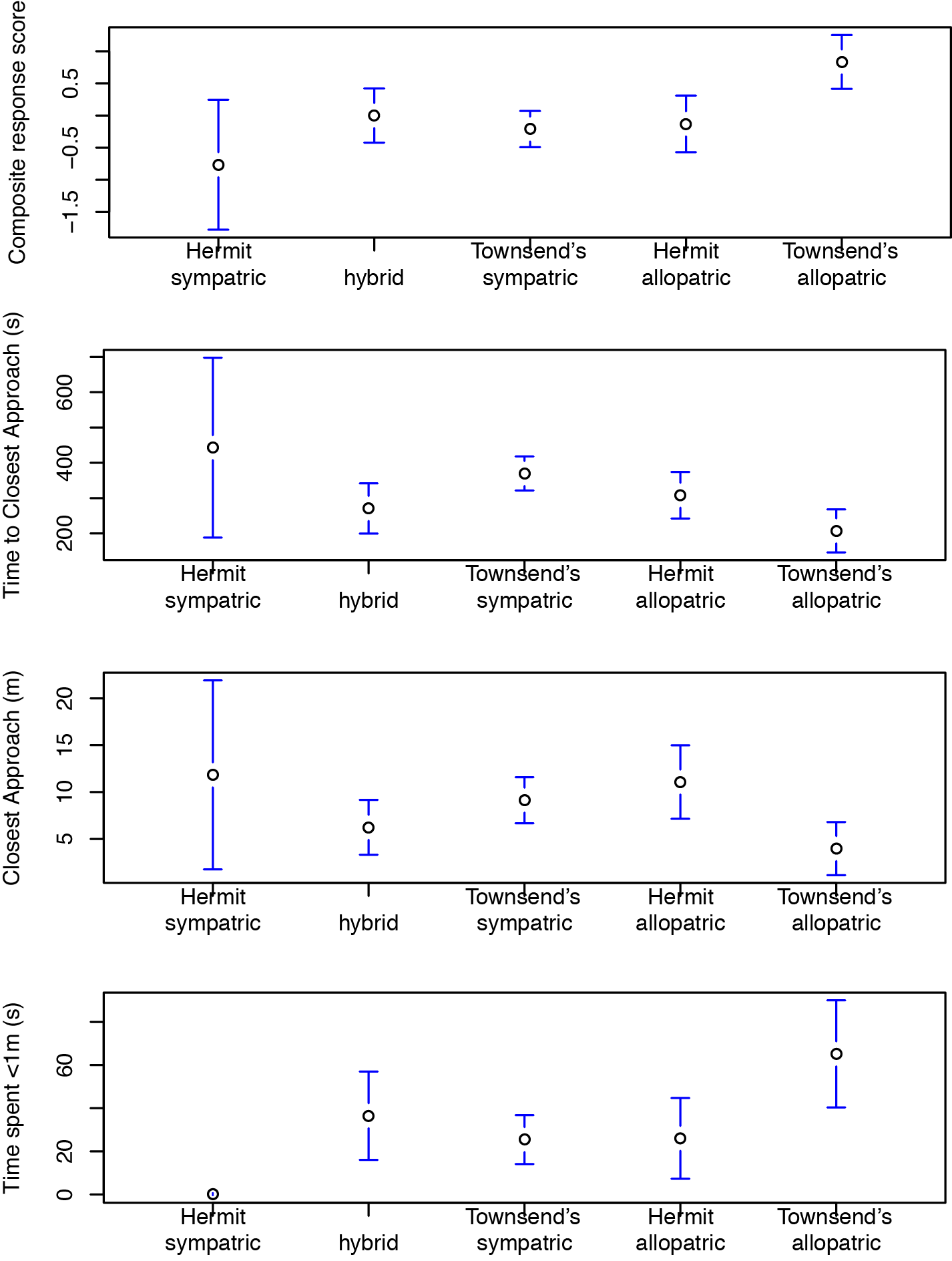


Figure S4. Composite response scores derived from PCA (top panel) reflect patterns observed in the raw response measurements (bottom three panels). Means+95%CI


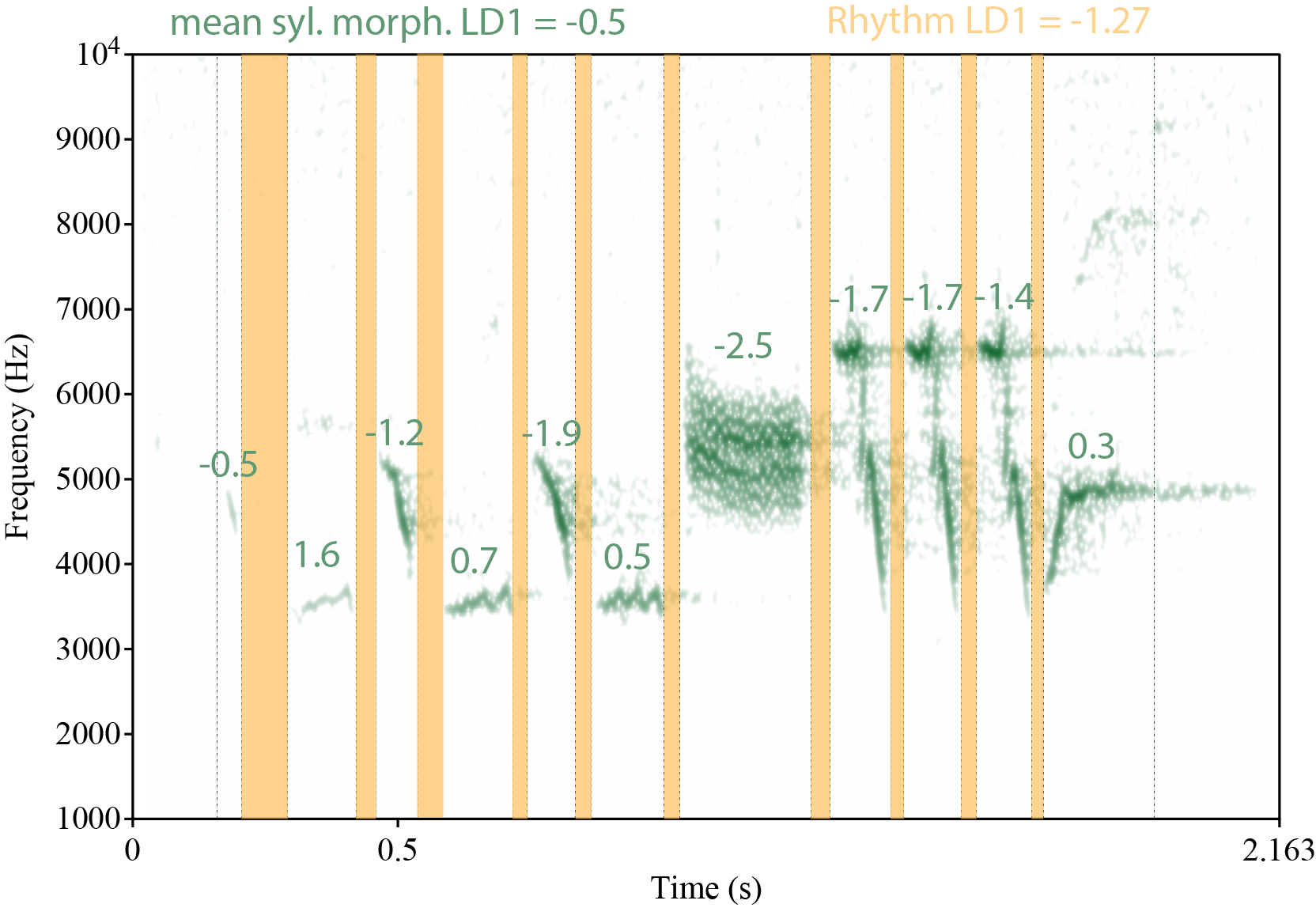


Figure S5. A song spectrogram showing song-average and syllable-level syllable morphology LD1 (green) and song rhythm (orange bands show silent periods, vertical dashed lines show start/end of syllables). Full details of measured features are included in the main text.
